# GAMT Deficiency: Clinical Presentation, Treatment, Diagnosis, Animal Models, Preclinical and Clinical Developments

**DOI:** 10.3390/ijms262311282

**Published:** 2025-11-21

**Authors:** Sara Biagiotti, Elena Perla, Serafina Manila Guzzo, Manuela Tolve, Francesca Nardecchia, Luigia Rossi, Claudia Carducci, Tiziana Pascucci, Vincenzo Leuzzi, Mauro Magnani

**Affiliations:** 1 Department of Biomolecular Sciences, University of Urbino, 61029 Urbino, Italy; sara.biagiotti@uniurb.it (S.B.); luigia.rossi@uniurb.it (L.R.); 2 Department of Psychology, Sapienza University of Rome, 00185 Rome, Italy; serafinamanila.guzzo@uniroma1.it (S.M.G.);; 3 Clinical Pathology Unit, AOU Policlinico Umberto I, 00161 Rome, Italy; 4 Department of Human Neurosciences, Sapienza University of Rome, 00185 Rome, Italy; francesca.nardecchia@uniroma1.it (F.N.);; 5 Department of Experimental Medicine, Sapienza University of Rome, 00185 Rome, Italy

**Keywords:** *GAMT* deficiency, creatine deficiency, *GAMT* gene, metabolic disorder, molecular pathology, diagnostics

## Abstract

Guanidinoacetate Methyl Transferase (*GAMT*) deficiency is a rare disease characterized by neurodevelopmental derangements, epilepsy, and movement disorders. The condition arises from the combined effect of postnatal brain creatine (Cr) depletion and guanidinoacetate (GAA) toxicity. Consequently, current treatment relies on Cr supplementation and metabolic management to reduce GAA accumulation by limiting its synthesis through ornithine supplementation and precursor reduction. Although effective in preventing the severe *GAMT* phenotype, the therapy is limited in normalizing these metabolites’ concentrations. Recently, interest has been growing in approaches aimed at restoring the mutant enzyme as the primary step toward a cure. Some of these approaches have been investigated at the preclinical level and are here summarized. Interestingly, a mouse model that replicates most of the patients’ features is now available in various labs, and the strong commitment of the Association for Creatine Deficiency has fostered the coordination and support of many of these models’ initiatives. This review introduces readers to the complexity of this ultrarare condition, describes current therapeutic approaches, provides information about the most accurate methods for an early diagnosis, and outlines the main features of the available animal models. Finally, some current preclinical investigations are described, along with some preliminary expectations of emerging data.

## 1. Introduction

Guanidinoacetate (GAA) methyltransferase (*GAMT*, EC 2.1.1.2) deficiency (MIM 601240) is a severe inherited disorder of Cr synthesis that results in postnatal neurodevelopmental impairment, developmental and epileptic encephalopathy and movement disorders. The condition is caused by the concurrent effects of prolonged cerebral Cr depletion and the accumulation of neurotoxic GAA. *GAMT* deficiency was first described in 1994 in 22-month-old boy patients presenting with developmental delay and progressive extrapyramidal symptoms, in whom brain proton magnetic resonance spectroscopy (H-MRS) revealed the absence of a cerebral Cr signal [1,2]. Reduced urinary creatinine excretion, reflecting a diminished total body Cr pool, serves as an additional metabolic indicator of this condition [3].

The biochemical markers of *GAMT*-D include increased GAA concentration, reduced Cr levels in bodily fluids (urine, plasma and CSF) and the absence of a significant reduction in the Cr peak at H1-MRS [4,5]. Urine GAA levels are approximately 1–30 times higher, plasma GAA levels are 2–20 times higher (with an average of 10 times) and CSF GAA levels are 5–50 times higher than the upper limit of the GAA reference range [6,7,8].

Diagnosis of *GAMT*-D is established by identifying biallelic pathogenic variants in the *GAMT* gene. Over 70 pathogenic variants have been reported in affected individuals, with approximately half classified as missense ones. Additional variant types include deletions, splice-site errors, frame shift, nonsense and truncating mutations [4,5,8,9]. Interestingly, in Portuguese patients, a high prevalence of the c.59G>C pathogenic variant indicates a possible founder effect in this region [10]. To date, approximately 130 cases of *GAMT*-D have been documented in the literature, either as individual reports or as small case series.

The estimated incidence of *GAMT* deficiency ranges from 1:2,640,000 to 1:250,000, with a reported incidence of 1:405,655 among newborns in Utah and New York [4,11,12]. These estimates align with newborn screening data, which indicated that over 1.5 million infants were screened, and two cases were confirmed [5,11,13,14,15,16]. The carrier frequency in the general population has been estimated at 1:372–1:812 [12].

## 2. Clinical Presentation

**Figure 1:**
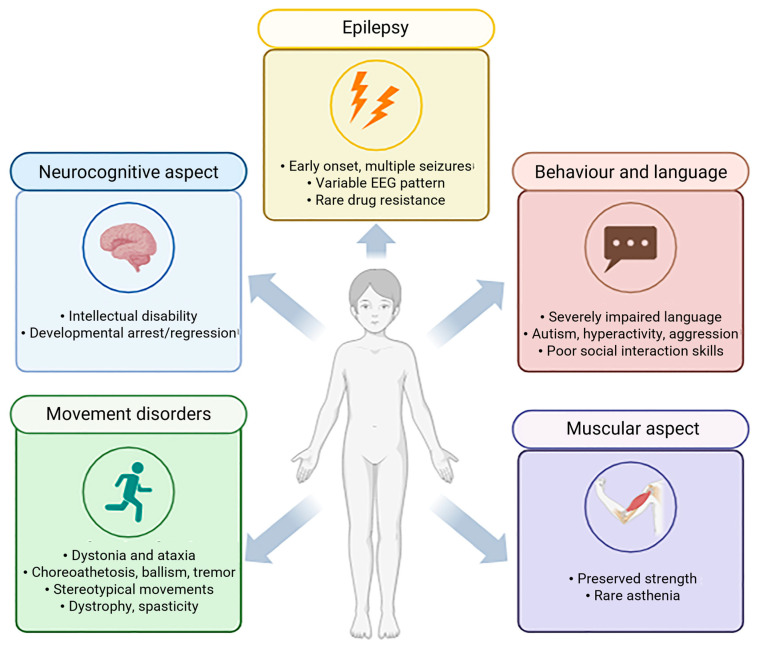
Clinical features of human *GAMT*-D patients. Main characteristics of neurocognitive, movement disorders, behaviour and language, muscular and epilepsy aspects (created with BioRender, https://www.biorender.com/).

Intellectual disability—the clinical hallmark of *GAMT* deficiency—is observed in all affected patients and, in most cases, is generally classified within the moderate to severe range (60–90%) [4,8,17] (Figure 1). Although less common, some patients may also experience developmental arrest or regression [4,18,19].

Epilepsy is the second most frequent manifestation of *GAMT*-D, which may be drug-resistant in a minority of cases [5,8,9,20]. In the initial phase of the disorder, febrile seizures commonly represent one of the earliest neurological manifestations, particularly between 3 and 6 months of age, whereas delayed onset forms are uncommon [21]. Even though no specific seizure pattern has been reported in *GAMT*-D, the affected patient may experience various types of seizures at different ages. Life-threatening tonic or myoclonic seizures may occur in the neonatal period, whereas myoclonic–astatic, generalized tonic–clonic, focal, drop attacks, atypical absences and staring episodes are more typical in infancy and childhood. Electroencephalography (EEG) findings are nonspecific and commonly reveal background slowing, multifocal spikes, slow-wave discharges and focal abnormalities in the frontal region [8,20].

The third most common feature is movement disorder, occurring in approximately half of patients—typically those with more severe phenotypes [8,17]. These manifestations are predominantly extrapyramidal and hyperkinetic. Dystonia and ataxia represent the most common forms, followed by chorea, choreoathetosis, ballismus, tremor and myoclonus. Stereotypic hand movements resembling those observed in Rett syndrome have also been described [22,23]. Bradykinesia and spasticity have been further associated with the condition [5,8,9,22,24]. Fever-induced ataxia, lasting several days and slowly remitting post-illness, has been noted in a single case [5]. Movement disorders typically emerge before 12 years of age [17], although later onset cases have been reported in a few individuals [25,26]. In most—but not all—patients with movement disorders, bilateral pallidal lesions have been identified on brain magnetic resonance imaging (MRI). These lesions typically appear as hypointense on T1-weighted MRI sequences and hyperintense on T2-weighted images, reflecting underlying tissue abnormalities [5,9,17,20]. Additional neuroimaging findings may include white matter alterations in the brainstem and pontine region, ventriculomegaly, cerebral atrophy and basal ganglia lesions [8,13,22].

In *GAMT*-D, a progressive course has been proposed, especially concerning motor features. In some late-diagnosed patients, worsening dystonia, tremor, spasticity and progressive dystonic–ballistic motor symptoms appearing in late childhood or adolescence have been reported, suggesting a possible neurodegenerative nature of the disease [8,17]. Nevertheless, the natural history of the disease remains poorly defined due to its rarity and the mitigating effects of early diagnosis and therapeutic intervention.

Language development appears profoundly compromised. Most patients never develop speech or acquire a very limited vocabulary, often producing fewer than 10 words [17,26,27,28,29,30]. To date, only one verbal patient has been reported in the literature—a 13-year-old girl, who was able to construct short and grammatically organized sentences [26].

Common features include poor or lacking eye contact, deficits in social communication and a lack of socio-adaptive skills, such as pointing or imitations [19,31,32]. Interestingly, only three patients have been described as having an unusually cheerful predisposition, with frequent episodes of laughter [27].

No specific muscular phenotype for *GAMT*-D has been consistently identified; however, muscle mass and strength are generally preserved, except for hypotonia [8,9,24]. An isolated case of asthenia [33] and descriptions of patients as slim but physically strong have been reported [23].

As for hearing, some authors reported mild hearing loss; however, it seemed to be reversible when treated with Cr supplementation and a restricted diet [9]. Other studies reported rare cases of bone deformities around the joints, particularly in the knees, legs and hands, leading to pain and difficulty in standing and walking short distances without support. These findings may reflect muscle atrophy, osteopenia or osteoporosis, potentially predisposing affected individuals to an increased risk of fractures [34].

## 3. Diagnosis of GAMT Deficiency

### 3.1. Biochemical Diagnosis

The levels of GAA and Cr in body fluids serve as reliable diagnostic markers for *GAMT* deficiency and should be used as first-line diagnostic tools [35]. Molecular genetic analysis and enzyme activity tests act as confirmatory methods. Since *GAMT* catalyzes the methylation of GAA into Cr, patients with *GAMT* deficiency usually present with significantly high GAA levels and low Cr concentrations [7,17,36].

Elevated GAA concentrations in plasma, urine and cerebrospinal fluid (CSF) represent the key biochemical hallmark of *GAMT*-*D* [5,22,37]. Affected patients show 4–130-fold increases in urine (relative to creatinine) and 4–22-fold increases in plasma [6]. GAA is both highly sensitive and specific for *GAMT*-D, though mild elevations may also occur in arginase deficiency [38].

Cr concentrations in plasma and urine are usually below normal in patients with *GAMT* deficiency, although in some cases, values may be only slightly decreased or even within the reference range [6,7]. The limited sensitivity of Cr as a biomarker may partly reflect dietary influences, especially with creatine supplementation [39]. However, assessing both GAA and Cr is recommended to provide a comprehensive overview of the metabolic pathway, as a combined analysis of these markers optimizes diagnostic reliability through improved sensitivity and specificity [39,40,41].

### 3.2. Reliability of Various Biological Fluids and Their Reference Values

Analysis of GAA and Cr can be performed in plasma, urine and CSF, as reported in Table 1. Plasma measurement is generally recommended as the primary approach for diagnosing *GAMT* deficiency [5,42]. However, the combined assessment of GAA in both plasma and urine enhances the robustness and reliability of the diagnostic evaluation [5,7].

A major limitation of urine analysis is the variability of analyte concentrations due to diuresis and renal function. Therefore, urinary metabolite values are commonly normalized to creatinine (Crn) [7]. This normalization aims to correct for differences in urine concentration, assuming that tubular excretion and reabsorption of these compounds are comparable. Moreover, Crn levels are directly influenced by Cr, which is generated by non-enzymatic conversion [43,44]. A reduction in Crn has been reported in *GAMT*-D, making the use of Crn for normalization suboptimal, as the Cr/Crn ratio may attenuate the degree of Cr reduction observed in affected patients [43,44]. This limitation is highlighted in the neonatal period, when *GAMT* deficiency patients may present a normal urinary GAA/Crn ratio [14,45]. For this reason, plasma GAA measurement is particularly important in this age group [36]. To optimize both diagnostic sensitivity and specificity, it is essential to establish a reliable method and population-specific reference values. Plasma and urine samples should be further collected from healthy individuals to define these normal ranges. The dependence of GAA and Cr on age has been evaluated both in plasma and urine. While the influence of age on plasma concentrations appears to be minimal, with inconsistent findings reported across studies, the impact on urinary values is considerably greater [46,47,48]. In healthy individuals, urinary GAA/Crn and Cr/Crn ratios decrease significantly with age [6,46,47,48]. Thus, age must be considered carefully when interpreting Crn-based ratios.

Most studies assessing GAA and Cr in *GAMT* deficiency report reference values; however, only a limited number included sufficient sample sizes for robust evaluation, particularly when age stratification is required. More recently, continuous age-adjusted reference limits for urinary Cr/Crn and GAA/Crn ratios have been established [49]. The obtained complex calculations and the resulting adjustments for age and other covariates were integrated into postanalytical interpretive tools for creatine deficiency syndrome (CDS) within the web-based Collaborative Laboratory Integrated Reports (CLIRs) platform [49] (https://clir.mayo.edu).

Reference and patient values may also be affected by sample conservation. Stability studies have shown that urinary GAA remains stable for at least 12 h at room temperature, whereas Cr is less stable, exhibiting an average increase of 27.1% over the same period [50]. In contrast, GAA and Cr in plasma and serum remain stable for at least 24 h at room temperature [50]. Consequently, samples should be frozen promptly, stored at −20 °C or lower, and transported on dry ice to ensure analytical reliability [50].

Finally, although CSF GAA levels in *GAMT*-D could even be 100–300 times higher than normal [17], CSF remains a challenging diagnostic specimen due to the invasive nature of sampling, which often requires sedation. In addition, baseline CSF GAA levels in healthy individuals are extremely low [6], demanding highly sensitive analytical methods [51]. The limited availability of normal control samples further restricts the establishment of reliable reference values, reducing the diagnostic utility of CSF in routine practice.

### 3.3. Methods for GAA and Cr Analysis

Increased GAA in patients with *GAMT* deficiency was first reported by Schulze et al. (1997), based on a quantitative analysis of 24 h urine using an ion-exchange chromatographic method [37,52]. Since then, several analytical methods have been described for the determination of GAA and Cr in plasma, urine, CSF and dried blood spot (DBS).

Method selection is guided by the available instrumentation—HPLC, GC–MS or LC–MS/MS—and the technical expertise within the laboratory. Method parameters such as linearity, precision, recovery, limit of quantification, turnaround time and sample preparation complexity must be considered when selecting a method [30,53,54,55,56].

The simplest methods are HPLC with a UV detector [56] and HPLC with fluorescence detection following derivatization with benzoin and chromatographic separation [7]. Despite being robust, simple, inexpensive and accurate for GAA analysis, the latter method cannot discriminate between Cr and Crn, since benzoin derivatization yields identical 2-substituted amino-4,5-diphenylimidazoles structures from both compounds [53]. As a result, Cr could only be calculated indirectly by subtracting Crn values [47].

With the increasing utilization of mass spectrometry (MS) in biochemical laboratories, methods coupling GC or HPLC/UPLC have been preferred over other methods [51].

The diagnostic reliability of GS-MS for *GAMT* deficiency has been demonstrated in several reports [6,46,55,57,58]. Although highly sensitive and accurate, this method is limited by lengthy and labour-intensive sample preparation, as both GAA and Cr are polar, basic molecules that require complex derivatization steps prior to GC analysis [55].

LC–MS/MS–based methods have proven highly reliable for the diagnosis of *GAMT* deficiency and are currently regarded as the method of choice. This approach combines high accuracy, precision and sensitivity with simplified sample preparation and suitability for high-throughput analysis [50,51]. Most of LC–MS/MS methods employ the esterification of carboxylic groups by HCl-butanol, followed by rapid chromatographic separation, positive electrospray ionization (ESI), multiple reaction monitoring (MRM) acquisition and quantification using stable isotope-labelled internal standards [59,60].

Alternative methods omitting esterification have also been described [50,51], providing the advantages of reduced sample preparation time and the possibility to measure Crn within the same analytical run. However, this benefit comes at the cost of reduced sensitivity, as omitting butylation results in a ~300-fold and ~60-fold decrease in signal intensity for GAA and Cr, respectively [54]. In addition, butylation facilitates the chromatographic separation of analytes. To address this constraint, several researchers have introduced ion-pair techniques as an alternative to enhance chromatographic resolution [61,62].

### 3.4. Newborn Screening and GAA Determination in DBS

Neonatal screening for *GAMT* deficiency is technically feasible and highly effective in preventing clinical manifestations [11,14,63]. Early diagnosis enables the prompt treatment, significantly improving outcomes and, in many cases, prevents the onset of neurological symptoms [64]. Broader implementation of screening in national programmes could greatly reduce the disability burden associated with this rare but treatable condition. Pilot initiatives in the U.S. have shown that *GAMT* deficiency can be reliably detected in the neonatal period [11].

The primary biochemical marker for the neonatal screening of *GAMT* deficiency is GAA in DBS. The initial detection of elevated GAA in neonatal DBS was achieved using high-performance liquid chromatography (HPLC) [53]. More recently, flow-injection analysis tandem mass spectrometry (FIA–MS/MS) methods for GAA quantification in DBS have become the gold standard for primary screening [11,14,65,66,67,68]. Although Cr measurement may be included in screening panels, Cr levels are often normal and are less reliable biomarkers than GAA. Consequently, GAA quantification in DBS remains the cornerstone of current protocols, supported by confirmatory plasma biochemical testing and molecular analysis of the *GAMT* gene [5,67].

Importantly, GAA measurement integrates into the existing tandem mass spectrometry-based multiplex platforms already in routine use for newborn screening, requiring no additional equipment and incurring minimal cost [69]. Both derivatized and non-derivatized methods have been proven successful [11,66]. Positive samples are characterized by elevated GAA concentrations and an increased GAA/Cr ratio [14]. The introduction of second-tier testing by HPLC–MS/MS [14], the application of *GAMT* gene sequencing directly on the first DBS [67] and continuous improvements in test reliability [68] have all contributed to enhancing specificity and ensuring high diagnostic accuracy in screening programmes.

Given the low expected case frequency, the relative simplicity of diagnostic confirmation and the substantial benefits of presymptomatic dietary therapy, *GAMT* deficiency was added to the United Stated Recommended Uniform Screening Panel (RUSP) in 2023. Extending newborn screening for this condition at the international level would represent an important and highly desirable public health objective.

### 3.5. Enzyme Activity Determination in Lymphoblasts, Fibroblasts and Lymphocytes

Assessments of *GAMT* activity have been established in both lymphoblasts and fibroblasts. Three main methods have been described. The first, reported by Ilas et al. (2000), uses a radiolabelled substrate to measure enzymatic activity [70]. Next, a non-radioactive method based on Cr measurement by high-performance liquid chromatography (HPLC) with UV detection was introduced [71]. The third method employs stable isotope-labelled substrates combined with gas chromatography–mass spectrometry (GC–MS) [72]. More recently, a quicker and minimally invasive approach has been developed, enabling direct measurement of *GAMT* activity directly in lymphocytes using LC–MS/MS. This approach overcomes the need for a labour-intensive cell culture to produce enough fibroblasts or lymphoblasts for enzyme confirmation [73]. Enzyme activity testing remains especially useful when biochemical marker levels and molecular genetic results do not match.

### 3.6. Molecular Genetic Analysis

The main diagnostic approaches are single-gene testing (sequence analysis followed by deletion/duplication testing if needed) and multigene panels including *GAMT*, *GATM* and *SLC6A8*; panel content and sensitivity vary across laboratories. Comprehensive genomic testing (exome or genome) is also feasible and does not require prior gene selection [28].

Thus far, a definitive correlation between genotype and phenotype has not been demonstrated. This is most likely due to the small number of patients reported worldwide and the wide distribution of private mutations across the gene [8] (https://www.orpha.net/en/disease/detail/382, accessed on 15 October 2025).

According to ClinVar (accessed on 6 September 2025), approximately 715 *GAMT* variants have been deposited. Of these, 268 are classified as variants of uncertain significance (VUS), 78 as likely pathogenic and 96 as pathogenic. Regarding molecular consequences, missense variants represent the most common class (n = 250), followed by frameshift (n = 52), nonsense (n = 26) and splice-site variants (n = 14).

In 2024, the ClinGen Cerebral Creatine Deficiency Syndromes Variant Curation Expert Panel (CCDS VCEP) established gene-specific variant classification guidelines for all three genes implicated in cerebral creatine deficiency syndromes, including *GAMT*. These guidelines were designed to promote consistency in variant interpretation and to resolve conflicts or outdated classifications in a systematic and standardized manner. The guidelines were created through a systematic review and refinement of the ACMG/AMP criteria for variant classification [74]. The specification of applicable codes was informed by both gene–disease-specific features and recommendations from the ClinGen Sequence Variant Interpretation (SVI) Working Group [75,76].

As of 23 June 2025, the ClinGen Variant Curation Expert Panel submitted about 110 *GAMT* variants to the ERepo database, an FDA-recognized database of human genetic variants that provides expert-curated classifications of pathogenicity. Each of these entries was assigned a three-star review status on ClinVar and supported by defined assertion criteria. Within this curated set, 23 variants were classified as benign or likely benign, 29 as variants of uncertain significance, 34 as likely pathogenic and 24 as pathogenic. Considering only the VUS, likely pathogenic and pathogenic categories, missense variants were the most frequently represented (n = 49), followed by frameshift variants (n = 13), nonsense variants (n = 12) and splice-site variants (n = 3).

The most common variants reported in individuals with *GAMT*-related CCDS were the following: (i) The synonymous variant NM_000156.6(*GAMT*):c.327G>A (p.Lys109=), identified in at least 30 unrelated patients with *GAMT* deficiency. RT-PCR and RNA sequencing analyses have shown that this variant produces two abnormal transcripts: one leading to the activation of a cryptic splice site in intron 2 and deletion of 146 bp, and another leading to a 44 bp insertion with altered splicing and skipping of exon 2 [77]; (ii) The missense variant NM_000156.6(*GAMT*):c.59G>C (p.Trp20Ser), found predominantly in Portuguese patients. Functional studies in HeLa cells demonstrated that this variant abolishes *GAMT* enzymatic activity despite normal protein expression, indicating that it produces a non-functional protein [10].

According to ClinGen guidance, any variant classified as likely pathogenic or VUS must be reassessed within 2 years to evaluate any new data that could change the classification [75,76].

## 4. Treatment

The treatment of *GAMT*-D aims to restore brain creatine and lower GAA levels [78]. The current metabolic therapy includes creatine supplementation (250–1000 mg/kg per day) combined with either a nutritional approach—such as low arginine intake [Arg 15/mg/kg body weight per day; proteins 1 g/kg body weight per day] and ornithine supplementation [100–800 mg/kg per day]—with or without a pharmacological strategy, including sodium benzoate and sodium phenylbutyrate [100 mg/kg body weight per day], to deplete GAA precursors and inhibit its synthesis. The effectiveness of sodium benzoate in depleting glycine and limiting GAA synthesis is debated; some support it use [79], while others contest it [80].

Regarding symptomatic subjects, the highest number of patients reported so far, current clinical outcome data come from cross-sectional or single-case retrospective reports of *GAMT*-D patients with heterogeneous ages and disease severity at diagnosis and treatment. Nevertheless, the treatment seems effective in improving seizures, movement disorders and, in young patients or those with milder presentation, neurodevelopmental issues [8,13,77,79,81,82].

Encouraging short-term follow-up results of a few early-treated presymptomatic young patients highlight the value of the early metabolic correction of Cr depletion and GAA intoxication in preventing *GAMT*-D phenotype development [11,29,83,84]. Similar outcomes in other neurometabolic disorders causing postnatal brain damage [85,86] further support *GAMT* deficiency as a candidate for neonatal screening [11].

Cr and GAA homeostasis in healthy individuals are highly regulated processes that balance endogenous Cr synthesis with nutritional intake to meet the ongoing needs of tissues such as muscle and brain tissue [87].

Despite promising early data, the long-term safety and effectiveness of the current treatment remain unclear. Additionally, the dosage of Cr supplementation and the target levels for brain Cr and GAA achieved through therapy are based on anecdotal evidence. H-MRS studies of treated patients indicated only partial recovery of brain Cr [79,88], while reaching normal levels of GAA in the blood and CSF appears difficult [11,79,83]. Additional safety concerns include the long-term tolerance of high doses of Cr supplementation and chronic brain exposure to persistently elevated GAA [12]. Lastly, as seen in other metabolic diseases [89], maintaining long-term compliance and treatment adherence can be challenging, especially for patients with *GAMT*-D who have severe intellectual disabilities and behavioural issues that may prevent them from following the treatment regimen [5,81,83].

## 5. Animal Models for GAMT Deficiency

### 5.1. The Use of Genetically Manipulated Animal Models in the Biomedical Research of Rare Diseases

Animal models have played a pivotal role in advancing biomedical science and improving human health and quality of life. Their use expanded markedly in the 20th century, and now spans nearly all fields of biomedical research. Among laboratory species, the mouse is the most used due to its genetic, anatomical, cellular, biochemical and behavioural similarities to humans. The presence of murine orthologs for most human genes, combined with practical advantages such as low maintenance costs, ease of handling and a short reproductive cycle, make mice especially suitable for long-term experimental studies [90].

In the early 1980s, for the first time, researchers successfully introduced modified DNA into embryonic stem cells [91]. This breakthrough enabled the genetic manipulation of organisms from the early developmental stages, leading to the creation of transgenic and knockout (KO) mouse models, that are now widely used in preclinical research.

The International Mouse Phenotyping Consortium has significantly contributed to this field by generating, characterizing and archiving over 6000 KO mice on the C57BL/6 background, the most well-known and widely used mouse [92]. Comprehensive databases of these genetically engineered models are publicly accessible through platforms such as the International Phenotype Mouse Consortium and the Jackson Laboratory (https://www.mousephenotype.org/, accessed on 15 October 2025).

Today, genetically modified mice are indispensable tools for modelling human pathological conditions, especially rare human diseases. These models facilitate the study of the underlying mechanisms and support the development of new therapeutic strategies.

### 5.2. The GAMT-KO Mouse Model

As with many rare diseases, systematic studies on the pathophysiology of *GAMT*-D in human subjects remain limited due to its low incidence. Therefore, to address this gap, genetically modified *GAMT* mouse models provide an effective approach for investigating this disorder.

The development of the *GAMT*-D genetic mouse model was first reported at the VIII International Conference on Inborn Errors of Metabolism (Cambridge, UK, 13–17 September 2000) [93]. Its subsequent validation to study Cr deficiency occurred three years later [94], followed by the characterization of tissue-specific *GAMT* mRNA and protein expression patterns in 2004 [95]. These studies clearly support the utility of the *GAMT*-KO mouse model for investigating *GAMT*-D.

The *GAMT*-KO mouse was generated by targeted disruption of the open reading frame within exon 1 of the murine *GAMT* gene in embryonic stem cells, resulting in a null allele. This genetic modification was confirmed at the DNA, RNA and protein levels [94]. Phenotypic characterization of *GAMT*-KO mice exhibited increased neonatal mortality, body weight reduction, muscular hypotonia, decreased male fertility and biochemical alterations similar to those observed in *GAMT*-D patients. These include a marked increase in GAA and reduced Cr and creatinine (Crn) levels in serum, urine and the brain. Furthermore, high levels of the phosphorylated form of GAA (PGAA) and reduced Cr phosphate (PCR) amounts in the heart, skeletal muscle and brain were detected using 31P MRS [95].

### 5.3. Biochemical Profiling

#### 5.3.1. Body Fluids

**Figure 2:**
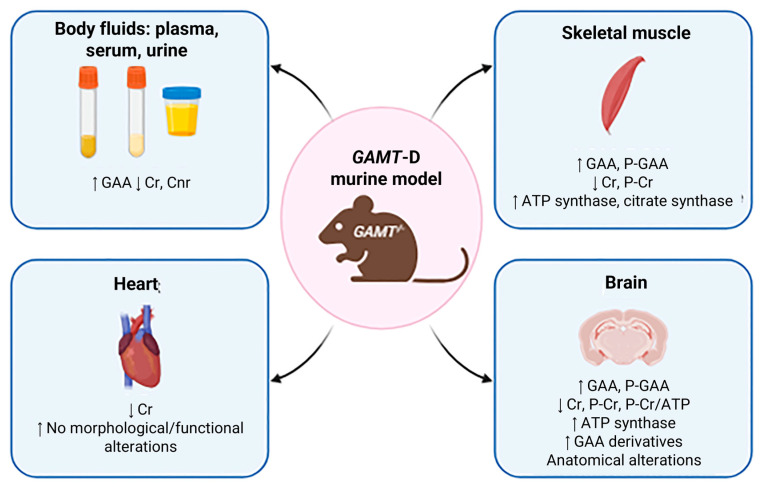
Biochemical profiling. Main features of *GAMT*-KO murine models are indicated by arrows. in the respective tissue, such as skeletal muscle, heart and brain or body fluids (created with BioRender https://www.biorender.com/).

In *GAMT* knockout (KO) mice, GAA concentrations were markedly elevated in plasma, serum and urine, while Cr and Crn were significantly reduced, mirroring the biochemical profile observed in humans with *GAMT* deficiency [95,96] (Figure 2). Wild-type (WT) and heterozygous (HZ) mice exhibited comparable profiles, with no notable differences detected in the evaluated parameters [3,7,97]. However, some discrepancies across studies underscore the necessity for standardized protocols and careful consideration of confounding factors such as dietary composition, breeding conditions and analytical techniques [95,96,98]. Notably, more recent investigations reported much higher GAA plasma levels (~1000 µM), emphasizing the critical importance of methodological validation. These aspects have been deeply examined in [99].

#### 5.3.2. Skeletal Muscle

*GAMT*-KO muscle is characterized by drastic GAA accumulation and Cr depletion, with phospho-guanidinoacetate (PGAA) replacing phosphocreatine (PCR) as an energy buffer, despite its lower efficiency [94,96,100] (Figure 2). In vivo ^1H and ^31P MRS, along with biochemical analyses, confirmed these changes. Moreover, other choline compounds were also detected by 1H-MRS, such as N-acetyl aspartate, taurine and lipids, except for PGAA, since it becomes visible on murine muscle as a broad line only when Cr is almost completely depleted [94].

Residual Cr observed in *GAMT*-KO muscle is likely due to dietary intake (Cr-free or not) or to coprophagy, if pups are not immediately separated from dams and housed individually by genotype [95,100]. Nonetheless, Cr levels are significantly lower than those in controls, supporting the depletion of high-energy phosphate in skeletal muscle. Interestingly, Renema et al. [94] reported an elevated PGAA/PCR ratio (3.4 ± 3), which can be explained by several mechanisms: (1) active GAA uptake via the creatine transporter, highly expressed in muscle and also capable of conveying GAA [87]; (2) endogenous GAA production via AGAT [101]; and (3) GAA use as an alternative substrate for creatine kinase (CK) [102]. Despite CK’s lower affinity for PGAA, this compound serves surprisingly well as an energy buffer in vivo, as demonstrated in a mild ischemic stress model [100]. Moreover, the accumulation of PGAA in GAMT-KO mice has emerged as a potential driver of reduced susceptibility to statin-induced muscle damage compared with AGAT-deficient mice [103]. In addition, both in vitro [100] and in vivo [95] studies showed no relevant alterations in ATP levels, supporting the presence of adaptations in mitochondrial function and oxidative phosphorylation.

Mitochondrial adaptations were evident, including increased ATP synthase and citrate synthase activity, with sex- and muscle-specific differences [95]. Compared with *AGAT* KO mice, the *GAMT*-KO phenotype was milder, possibly due to PGAA’s compensatory role and its effect on AMP-K signalling, which may prevent severe muscle atrophy. These findings align with human *GAMT*-D muscle profiles [97,102].

#### 5.3.3. Heart

Due to the well-known role of Cr in tissues with high energy demand, several studies over the last 20 years have examined cardiac energy metabolism in *GAMT*-KO mice (Figure 2). In 2004, Schneider and colleagues [104] achieved the application of cardiac 1H-MRS in in vivo wild-type and *GAMT*-KO mice and showed the absence of the creatine-methyl resonance at 3 ppm, confirming Cr deficiency in the mouse model of *GAMT*-D. In 2005, the authors studied the cardiac phenotype of male and female *GAMT*-KO mice, in vivo (MRI) and invasively (perfused heart), measuring cardiac function at low workload and under stress by inotropic stimulation and ischemia/reperfusion [105]. Observations indicate that GAMT-KO mice do not develop cardiac hypertrophy and maintain normal function under low-workload conditions, suggesting compensatory metabolic adaptations. However, under stress-induced physiological demands, these mice exhibit an abnormal cardiac phenotype, highlighting latent vulnerabilities in energy metabolism. In 2008, ten Hove and colleagues studied cardiac Cr uptake in isolated hearts from two mouse models of altered myocardial Cr levels: *GAMT*-KO and CrT-OE (overexpression of myocardial CrT) mice, in ^14^C-radiolabeled Cr perfused hearts. In *GAMT*-KO hearts, the authors observed significant increases in creatine uptake and in Cr transporter mRNA levels [105]; these increases were prevented by a Cr-supplemented diet, suggesting a negative feedback regulation of Cr uptake. Furthermore, Lygate and colleagues demonstrated in male *GAMT*-KO mice that chronic Cr deficiency is compatible with survival post-myocardial infarction [106].

In 2008, other researchers published a one-year longitudinal MRI study on male and female *GAMT*-KO mice, collecting measurements of cardiac structure and function at 6 weeks, and at 4, 8 and 12 months of age [107]: the study suggested again the presence of compensative adaptations in the *GAMT*-KO mice able to counteract the effects of the creatine lack on the heart structure and function, remaining normal during ageing. Between 2019 and 2020, authors published results from observations of cardiac function in *GAMT*-mice beyond 1 year of age [108]: function, metabolism and mitochondrial organization were reported in *GAMT*-KO hearts, confirming previous data showing that Cr-deficiency does not directly impair heart function and suggesting the presence of compensatory strategies in ageing hearts. Branovets and colleagues determined the expression of alternative energy transfer systems, hexokinase (HK) and adenylate kinase (AK) functions, in male and female creatine-deficient AGAT-KO and *GAMT*-KO mice [109]: the authors did not find changes in the activity and mitochondrial association of AK, or HK in hearts from *GAMT*-KO mice (whereas some effects have been observed in the AGAT-KO model).

The effects of gene therapy using adeno-associated virus expressing the human *GAMT* gene in *GAMT*-KO mice have been investigated by Khoja and colleagues [110], which reported normalization of Cr levels in the heart (as well as in the brain, kidney, liver and skeletal muscle) of *GAMT*-KO treated mice.

#### 5.3.4. Brain

The identification of primary creatine deficiency syndromes (CDS), including *GAMT* deficiency, has clarified the critical role of Cr metabolism in brain development and function (Figure 2). In *GAMT*-KO mice, cerebral metabolic alterations closely mirror those observed in human patients. ^1H and ^31P MRS revealed a profound depletion of brain Cr and PCR, accompanied by elevated GAA and reduced PCR/ATP ratios. PGAA was also detected, though at lower levels than in muscle [94].

Despite severe Cr depletion, ATP levels remained stable, supported by compensatory increases in mitochondrial ATP synthase activity (~80%) [111]. Additionally, several guanidine derivatives—such as GSA, ArgA, β-GPA, γ-GBA and homoarginine—were elevated in the brains of *GAMT*-KO mice [96]. Some biochemical discrepancies with human CSF (e.g., absence of arginine depletion, higher GSA in mice) may explain the milder neurological phenotype in mice, which lacks seizures or ataxia [95].

PGAA may contribute to neuroprotection, acting as an alternative energy buffer, and its accumulation has been reported in both *GAMT*-KO mice and patients [88,97]. Furthermore, recent studies using RNAi-induced partial *GAMT* deficiency in 3D rat brain cultures showed that even moderate GAA accumulation disrupts neuronal development and cell survival [112].

Finally, structural MRI revealed abnormalities in several brain regions of *GAMT*-KO mice, supporting a role for Cr depletion in neuroanatomical changes [113]. Moreover, recent studies have exploited the role of myelin in rare genetic diseases characterized by brain injury; hence, it could be stimulating to deepen this aspect in *GAMT*-D mice models also [114].

### 5.4. Clinical Characterization of GAMT-KO Mice

#### 5.4.1. Early Postnatal Development: Reduced Body Weight

**Figure 3:**
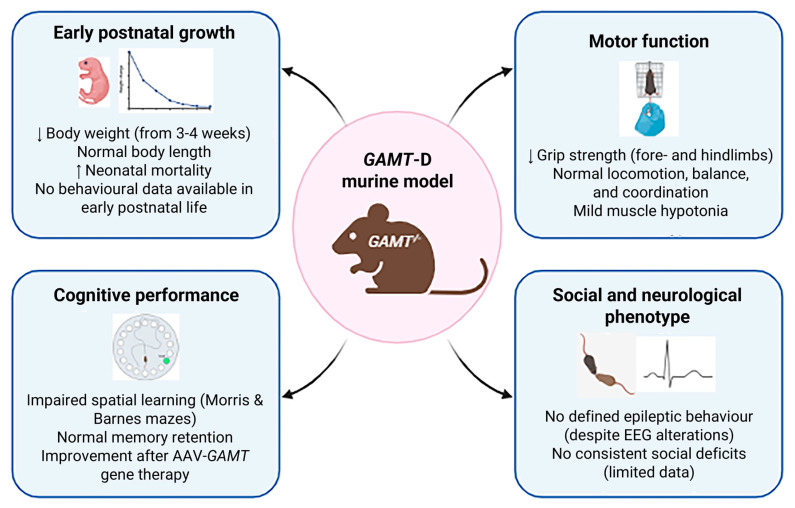
Phenotypic characterization. Main behavioural and developmental features of *GAMT*-KO murine models are indicated by arrows, including growth, motor, cognitive and social/neurological profiles (created with BioRender https://www.biorender.com/).

Studies on *GAMT*-KO mice have demonstrated a faithful reproduction of the biochemical profile of the human disease (GAA accumulation and Cr reduction in various tissues—brain, muscle, heart—and body fluids—serum, urine). Regarding physical development, several articles reported reduced body weight (due to reduced body fat mass and muscle hypotonia) in male and female *GAMT*-KO mice in comparison with wild-type controls [95] (Figure 3). In particular, the weight of *GAMT*-KO mice is comparable with control littermates at birth, decreases starting from 3/4 postnatal weeks and worsening with time (measured until 24 months of age by Smith and colleagues), and the difference is more pronounced in female *GAMT*-KO mice. Although *GAMT*-KO appeared lighter, no significant body length differences were found [95].

Although *GAMT*-D is a metabolic neurodevelopmental disorder, to the best of our knowledge, no data are still available today on the behavioural phenotype of *GAMT*-D mice during early postnatal life, as highlighted in our previous review [99]. The only behavioural data available are in fact carried out on adult animals [95,96,98,100,106], and studies investigating early-onset behavioural deficits in *GAMT*-KO mice are not present.

Preliminary observations from our laboratory on the early postnatal period (P8–P17) indicate that the acquisition of basic sensorimotor reflexes, such as righting, screen and auditory startle, appears delayed in *GAMT*-KO pups compared with wild-type littermates. These findings, while preliminary, point to a developmental delay possibly linked to early creatine deficiency and warrant longitudinal analyses to assess its impact on subsequent motor and cognitive maturation.

#### 5.4.2. Movement Assay: Compromised Motor Grip Force

Physiological Cr levels are important for psychomotor development, and reduced Cr in *GAMT*-D causes biochemical alterations in the skeletal muscle; therefore, several studies have explored movement disorders in *GAMT*-KO mice [99] (Figure 3).

*GAMT*-KO mice exhibit lower motor grip force than wild-type when submitted to the grip strength meter. A reduced grip strength on the forepaw and back paw has been reported [95,110,115]. Only this motor activity results in compromised *GAMT*-KO mice.

A complete evaluation of motor analysis has been performed for the first time by Torremans [96], through ambulatory cage activity (2 h in dusk phase and 16 h overnight), swim activity (during Morris water maze), equilibrium and motor coordination (on accelerating Rotarod test) and locomotor activity (in Open Field test). In none of these tests did the authors report significant differences between *GAMT*-KO and wild-type mice, observing no differences between the groups in 2 h and 16 h activity in the home cage; swimming velocity in the Morris water maze; neuromotor performance in the accelerating Rotarod test; or distance moved in the Open Field test.

The only exception concerns the observation that, in the Open Field test, *GAMT*-KO mice entered corners less than heterozygous mice, but the authors themselves acknowledge that this observation is difficult to interpret. Furthermore, Iqbal and colleagues [98] investigated the effect of 2% Cr monohydrate supplementation in female *GAMT*-KO mice in the Rotarod and Open field, demonstrating the effect of Cr supplementation only in the performance on the Rotarod test (*GAMT*-KO mice supplemented with 2% Cr resisting on rotating apparatus for a longer time).

Lygate and colleagues [106] evaluated voluntary exercise (in running wheels in the home cage) and forced exercise (treadmill with electric shocks from the grid) in *GAMT*-KO and wild-type female mice, observing comparable voluntary running and average or maximum speed in the running wheel placed in the home cage for 3 weeks. Moreover, Cr supplementation did not modify running performance in *GAMT*-KO mice, suggesting that voluntary running is unrelated to creatine deficiency. Unexpectedly, *GAMT*-KO mice ran faster than WT on the treadmill.

#### 5.4.3. Cognitive Assay: Delay in Spatial Information Processing

Since intellectual disability is the clinical hallmark of *GAMT*-D, present in all affected patients, *GAMT*-KO mice are submitted to several cognitive tests.

One of the most complete studies of cognitive performance in *GAMT*-KO mice was performed by Torremans et al. [96]. Control, KO and HZ *GAMT*-D male mice from the age of six months were subjected to the Morris water maze. Although no different performances were reported between groups during the training phase, nor in swimming velocity during the test trial, *GAMT*-KO mice spent significantly less time in the target quadrant containing the platform (data confirmed by [98]) compared to healthy and heterozygous mice (but the frequency of entries to this quadrant did not differ between groups). Torremans et al. [96] also explored learning and memory performances in the passive avoidance task, reporting no significant differences between groups (Figure 3).

In 2022, Khoja and colleagues [110] submitted *GAMT*-KO mice to the Barnes maze, a hippocampal test that measures the mouse’s ability to learn the relationship between environmental distal cues and a fixed escape location, and observed a deficit during the acquisition phase (*GAMT*-KO mice acquiring the ability more slowly and with a longer distance), whereas short- and long-term memory remained unaffected. Furthermore, the authors demonstrated the recovery of the acquisition phase performance following an AAV-based *GAMT*-gene therapy.

Preliminary data from our laboratory using the Spatial Novelty Test appear consistent with these findings, suggesting that *GAMT*-KO mice display reduced spatial exploration of novel environments, in line with the spatial learning deficits previously described in hippocampus-dependent tasks.

#### 5.4.4. Social Assay: Absence of Evidence

Although several autistic-like features are frequently reported in the *GAMT*-D (absence of active speech, hyperactivity, aggressive behaviour, self-injurious behaviour, excessive interest in objects over social interaction and stereotypic behaviours), only one study summarily investigates social behaviour in *GAMT*-KO mice [96]. Authors hurriedly reported no significant differences between *GAMT*-KO and control mice in the social exploration test, measured by putting two females in the centre of the square Open Field test (Figure 3).

As reported in our preview review, the absence of a systematic evaluation of social behaviours in *GAMT*-D mouse model remains.

## 6. Clinical and Preclinical Developments

### 6.1. Clinical Developments

Therapeutic strategies aimed at normalizing cerebral Cr levels in *GAMT* deficiency primarily involve oral administration of high-dose Cr monohydrate (400–600 mg/kg body weight) [77], often in combination with ornithine supplementation (100–800 mg/kg/day given in 3–6 doses daily) and/or dietary arginine restriction (15–25 mg/kg/day). These interventions are designed to reduce GAA accumulation, which is neurotoxic at elevated concentrations (Creatine Deficiency Disorders—GeneReviews^®^—NCBI Bookshelf). Management of *GAMT* deficiency required regular monitoring of plasma GAA and ornithine levels to guide treatment efficacy and safety. Clinical improvements are typically observed in seizure control, behavioural regulation and motor function; however, intellectual disability and developmental delays present prior to diagnosis are generally irreversible. Therefore, early detection remains critical to preventing permanent neurological damage (Orphanet: Guanidinoacetate methyltransferase deficiency). As for the September 2025 query, ClinicalTrials.gov and EU Clinical Trials Register for “*GAMT* Deficiency” did not match any clinical trials.

### 6.2. Preclinical Developments

From the biochemical point of view, *GAMT* catalyzes the last step of Cr synthesis, facilitating the transfer of a single methyl group from S-adenosyl methionine (SAM) to GAA, forming creatine and S-adenosylhomocysteine (adoHcys). Cr is mainly synthesized in the liver of all mammals, released into the blood and captured by the tissues that require it. At the biochemical level, the disease is characterized by extremely low levels of Cr in the tissues and bodily fluids (urine, plasma and CFS), and by the accumulation of its precursor GAA, a highly toxic molecule for the organism, especially for the brain [17]. Thus, to be successful, the therapy must consist in lowering the high levels of circulating GAA and restoring normal levels of creatine in the brain, muscle and other tissues. Throughout the administration of synthetic *GAMT*, the defective enzyme, in a cellular context, is capable of providing SAM as a methyl donor. Magnani et al. [116] have provided evidence that human red blood cells (RBCs) metabolically engineered with the encapsulation of the recombinant enzymes *GAMT* and methionine adenosyl transferase (*MAT*) perform in vitro as competent bioreactors to reduce GAA and produce Cr, thus representing a potential strategy to treat patients with *GAMT* deficiency. Enzyme replacement therapies are commonly adopted for treating many rare diseases, i.e., [117,118,119,120]. However, no evidence is available for the engineering of an entire metabolic pathway (recombinant human *GAMT* plus recombinant *MAT*) as a potential strategy to treat patients with *GAMT* deficiency, capable of reducing the high toxic levels of GAA and restoring proper Cr levels in blood and tissue. It is worth noting that the production of recombinant enzymes could generate proteins with limited stability and frequently low solubility. The production of the human recombinant *GAMT* is not an exception. The first problem encountered was due to a limited stability of a recombinant *GAMT* with the loss of activity already after only a few days at 4 °C preventing any possible preclinical experiment. Furthermore, solubility limited the protein function and the possibility of scaling up the production process. Mutagenesis of four critical amino acids in the wild-type enzyme was necessary and was able to increase both the solubility and the stability of the recombinant protein over time [116]. Encapsulation of this enzyme in human RBCs provided evidence that a functional conversion of GAA into Cr also requires the co-encapsulation of *MAT* (EC:2.5.1.6). This approach was successful in generating strong evidence for the engineering of an entire pathway able to catalyze the conversion of GAA into Cr in human cells. The key requirements for this successful approach were strictly dependent on the engineering of human wild-type recombinant *GAMT* by the substitution of four critical amino acids and the simultaneous presence of a recombinant *MAT* consisting only of the human isoform *MAT2A* (human cells can express three different *MAT* isozymes [121], excluding the presence of the regulatory subunit *MAT2B*). The experience gained during this work provided the bases for starting a new programme consisting in the development of a new delivery system no longer based on autologous red blood cells engineered by the encapsulation of r*GAMT* and r*MAT* but by RBC-derived extracellular vesicles (RBCEVs) loaded with *GAMT* mRNA coding for the mutagenized proteins developed by site-specific mutagenesis, as described above [122]. Preliminary data have been presented at the 2025 CDC virtual conference on 23 August (2025 Virtual Conference High Level Agenda).

### 6.3. Adeno-Associated Virus-Based GAMT Gene Therapy

Adeno-associated viral vectors of different serotypes are commonly used in gene therapy [109]. Gerald S. Lipshutz and colleagues engineered an AAV serotype rh10 vector expressing human codon-optimized *GAMT* (hco*GAMT*) under a liver-specific (thyroxine-binding globulin [TBG]) promoter to treat *GAMT* deficiency mouse models. Genome copies (GC/Kg body weight) tested ranged from 5 × 10^12^ up to 1 × 10^14^. *GAMT* mRNA and liver protein expression increased with the number of genome copies administered [110]. At the same time, an improvement in metabolic response was evident, reaching plasma Cr levels equivalent to the mouse WT, while GAA was also reduced and normalized at the highest dose tested (10^14^ GC/Kg). Treated animals were followed for one year. The data collected demonstrated a marked improvement in cerebral and myocardial creatine levels, near normalization of weight and normalization of some behaviour alterations. PET-CT imaging demonstrated improvement in brain metabolism.

The experiment also provided data to be considered for further investigations, especially from a safety point of view. High levels of antibodies against AAV rh10 were detected in all treated animals, as expected when similar adeno-associated viral vectors are used. In terms of efficacy, by 1 year, AAV-treated *GAMT*^-/-^ female mice had substantial weight gain and were comparable to WT. On the other hand, male mice demonstrated substantial weight gain when compared with untreated *GAMT*^-/-^ mice, but not to the same extent as the WT controls. AAV genomes were retained in the liver of treated mice at 54 weeks from administration (the last time point investigated), even though the number of copies was significantly reduced. The treatment was able to restore the physiological Cr level in all tissues investigated, namely brain, kidney, muscle, heart and liver. Fluorodeoxyglucose (18F-FDG)-positron emission tomography (PET) demonstrated that glucose uptake was normalized in the brains of treated animals. In conclusion, the gene therapy approach with a specific targeting to the liver resulted in the normalization of GAA and increased creatine with limited side effects in the investigated period. Furthermore, these data support the concept for considering liver as the main source of Cr from GAA, almost normalizing these metabolites in the different tissues investigated.

Although the report from Gerald S. Lipshutz and colleagues resolves most of the deficiencies of the *GAMT*^-/-^ mice model and provides evidence for a reduced GAA in the brain, the figures do not support a complete normalization of this toxic metabolite in the treated animals [110]. To solve this problem, other authors have recently presented a CNS-directed gene therapy aiming at normalizing also these toxicities. J.S Walia and colleagues attempted especially to treat the high level of GAA and the low level of Cr in the brain, and have developed a different vector known as AAV9 and used a ubiquitous promoter [123]. This viral vector is expected to cross the BBB and directly deliver the gene to the CNS. To further improve the efficacy in terms of preventing inactivation by neutralizing antibodies and lowering genome copy numbers, the administration via an intrathecal route was preferred. Treated animals were followed for 8 weeks, and an increased amount of Cr in serum over time and a decrease in GAA were documented; however, it did not reach the level of WT mice. It is worth noting that biodistribution analysis showed the highest copy number of h*GAMT* DNA in the liver, followed by the lumbar spinal cord (the site of vector administration), followed by the heart, lung, spleen and kidney. Sections of the brain (caudal and rostral) had the lowest content of h*GAMT* DNA. In summary, this preclinical investigation, although delivered via the intrathecal route, does not normalize GAA in the brain, nor does it restore creatine in the cerebral cortex and cerebellum. It is not clear if, to prevent the toxicity by GAA, its concentration should be within the physiological range in WT mice in the serum and brain, or if a significant reduction is sufficient. This and additional considerations suggest that the experiments described above do not clarify the issue and require further investigation.

In conclusion, while several preclinical investigations have been conducted in animal models (not discussed in this paper), no additional data have been reported, except those described here.

## 7. Conclusions

*GAMT*-D is a severe and extremely rare metabolic disorder, although the possibility of a higher, underestimated prevalence should be considered. Current treatments show clear benefits when started before symptoms appear, but long-term outcomes remain uncertain. Including this disease in newborn screening panels is therefore essential for early diagnosis and treatment of affected children, while ongoing preclinical research is vital to improve therapies and enhance lifelong results. In this context, genetically manipulated animal models—and particularly the *GAMT*-KO mouse—represent indispensable tools for elucidating the molecular and pathophysiological mechanisms of rare diseases and for accelerating the development of targeted therapeutic strategies.

The biochemical and functional profile of the *GAMT*-KO mouse faithfully recapitulates the human disorder, revealing tissue-specific compensatory adaptations (e.g., PGAA accumulation and mitochondrial remodelling) that account for the relative preservation of certain physiological functions and underscore the model’s translational relevance.

Nevertheless, significant knowledge gaps persist—most notably the absence of a defined epileptic phenotype despite reported EEG abnormalities, together with the limited investigation of early postnatal development and social behaviours—highlighting the need for standardized behavioural protocols, longitudinal and interventional studies and integration with multi-omics approaches to achieve a comprehensive phenotypic characterization and to guide future therapeutic innovations.

## Figures and Tables

**Table 1:** Summary of *GAMT*-D diagnosis methods.

DIAGNOSIS METHOD FOR *GAMT*-D		
**TYPE OF SAMPLE**	**PROS**	**CONS**
**PLASMA**	GAA and Cr stability up to 24 h, no age-influenced	
**SERUM**	GAA and Cr stability up to 24 h	
**URINE**	More robustness for diagnosis	Influenced by renal function, instability both GAA and Cr over time
**CFS**	Highly sensitive	Invasive sampling, nearly undetectable GAA in healthy individuals
**DRIED BLOOD SPOT (DBS)**	Newborn screening: improved diagnosis	

## Data Availability

No new data were created or analyzed in this study.
